# Nonrestorative Sleep and Type 2 Diabetes Incidence: The Aichi Workers’ Cohort Study

**DOI:** 10.2188/jea.JE20230184

**Published:** 2024-09-05

**Authors:** Jingyi Lin, Zean Song, Yuanying Li, Chifa Chiang, Yoshihisa Hirakawa, Yoshihisa Nakano, Young-Jae Hong, Masaaki Matsunaga, Atsuhiko Ota, Koji Tamakoshi, Hiroshi Yatsuya

**Affiliations:** 1 Department of Public Health and Health System, Nagoya University Graduate School of Medicine, Nagoya, Japan; 2 Department of Public Health, Fujita Health University School of Medicine, Toyoake, Aichi, Japan; 3 Department of Nursing, Nagoya University Graduate School of Medicine, Nagoya, Japan

**Keywords:** nonrestorative sleep, diabetes, cohort study, Japanese

## Abstract

**Background:**

The term “nonrestorative sleep (NRS)” refers to an unrefreshed feeling at wake-up and is a domain of poor sleep quality. Previous research has demonstrated that NRS is linked to a number of diseases and adverse health outcomes, but less is known regarding the link between NRS and diabetes, particularly in Japanese.

**Methods:**

We studied 3,665 middle-aged male participants of the Aichi Workers’ Cohort Study who were followed-up from 2002 through 2019. Cox proportional hazards models estimated hazard ratios (HRs) and 95% confidence intervals (CIs) of incident type 2 diabetes mellitus (T2DM) in relation to NRS adjusted for potential confounding variables.

**Results:**

During a median follow-up of 14.6 years, 421 type 2 diabetes cases were identified. Participants with NRS had a higher crude incidence rate of T2DM (11.2/1,000 person-years), compared to participants without NRS (9.3/1,000 person-years). In the fully adjusted model, individuals who reported having NRS had a significantly higher risk of developing T2DM (HR1.36; 95% CI, 1.10–1.67). The association was observed only in participants under 50 years old (HR 1.82; 95% CI, 1.36–2.43), not in the older (50 years or older) participants (*P* for interaction = 0.025). In contrast, stratified analyses by the presence of shift work, obesity, or sleep duration showed similar associations in all the strata.

**Conclusion:**

NRS was associated with higher risk of T2DM in middle-aged Japanese male workers independent of a variety of lifestyle factors and other sleep problems.

## INTRODUCTION

The prevalence of diabetes has been rising rapidly globally, fueled by the population aging and increases in obesity.^1^ In 2019, diabetes was the direct cause of 1.5 million deaths, 48% of which occurred before the age of 70 years according to World Health Organization. A variety of lifestyle factors are related to the development of type 2 diabetes mellitus (T2DM), such as diet, physical activity, and smoking.^2^

Sleep is a biologically important reversible state of inactivity associated with reduced responsiveness to the external environment.^3^ Approximately one-third of our day consists of sleep. However, more people are suffering from sleep problems in modern culture as a result of work stress, shift work, spending time on electronics before bed, drinking coffee, and other behaviors. Sleep may also play a role in the development of T2DM as a previous systematic review that included 10 studies reported that quantity (both short and long sleep) and quality (insomnia symptoms) of sleep were significantly related to T2DM incidence.^4^

Nonrestorative sleep (NRS) refers to experiencing an unrefreshed feeling upon awakening, recognized as one of the sleep problems.^5^ Several articles reported that NRS was related to some adverse health outcomes and diseases. For example, in the Nagahama Study, NRS was significantly associated with gastroesophageal reflux disease symptoms, depressive mood, and nocturia symptoms.^6^ A meta-analysis indicated that NRS was related to a 16% higher risk of first-ever cardiovascular diseases incidence during the follow-up.^7^ However, the impact of NRS on incident diabetes has only been examined in a small number of longitudinal studies. A 5-year community-based follow-up study mentioned the long-term NRS was significantly associated with T2DM (odds ratio [OR] 2.63; 95% confidence interval [CI], 1.23–5.63).^8^ After adjusting for age, gender, educational level, marital status, family income, regular use of medications, and other subtypes of insomnia, the association between NRS and T2DM (OR 3.72; 95% CI, 1.66–8.36) remained significant even after excluding short sleepers in that study. Another longitudinal study conducted in Japanese population found that NRS was significantly associated with the risk of T2DM (hazard ratio [HR] 1.06; 95% CI, 1.00–1.12) after adjusting for age, gender, smoking, heavy alcohol consumption, skipping breakfast, and non-regular exercise.^9^ Japan’s prior research on NRS and T2DM has been limited, but our approach is more comprehensive, accounting for a broader spectrum of covariates. We have also conducted stratified analyses and extended follow-up periods.

Considering the potentially wide-spread public health implications of NRS, this study was established to investigate whether NRS is related to incident T2DM in middle-aged Japanese working men of the Aichi Workers’ Cohort Study, while taking into account potential covariates like perceived stress, baseline body mass index (BMI), and fasting blood glucose (FBG).

## METHODS

### Study population

**Figure 1.**
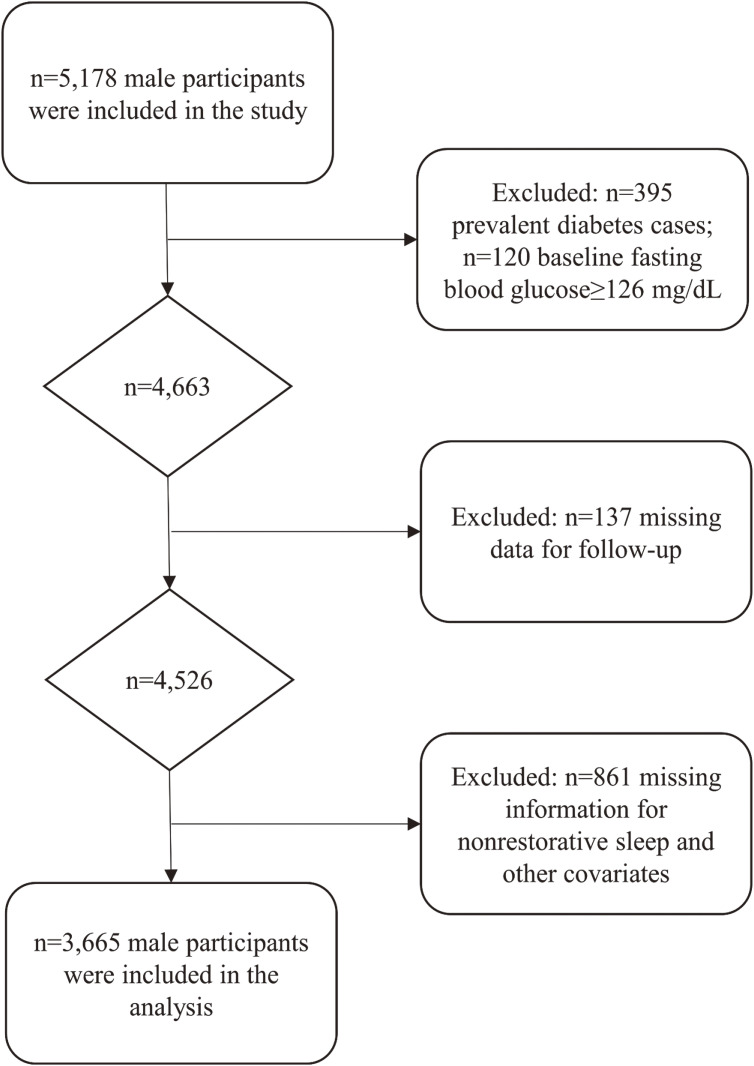
Exclusion flow chart

Strengthening the Reporting of Observational Studies in Epidemiology (STROBE) guidelines were followed in the conduct and reporting of the study. Data of the Aichi Workers’ Cohort Study, an ongoing study on non-communicable diseases was used. The study included 6,648 Japanese workers in a worksite, ranging in age from 35 to 66 years. Baseline data was gathered in 2002 using self-administered lifestyle and medical history questionnaires, and an obligatory annual health checkup offered by the employer. Since the number of female participants who developed T2DM during the follow-up was small, we restricted the present analysis to men (*n* = 5,178). We then excluded the following individuals (Figure 1): (1) prevalent cases of diabetes mellitus (*n* = 395) identified using self-reported medication use or baseline fasting glucose level ≥126 mg/dL (*n* = 120); (2) those with missing data for follow-up (*n* = 137); (3) those with missing information for NRS (*n* = 102); and (4) those with missing information for other covariates (*n* = 759) (BMI: *n* = 249; physical activity: *n* = 178; perceived stress: *n* = 112; sleep duration: *n* = 129; shift work: *n* = 91). The analytic sample for this study included 3,665 Japanese male workers who were at risk for developing diabetes during the follow-up.

Every person was tracked until the ascertainment of diabetes, censoring, or the end of the follow-up period (March 2019), whichever occurred first. Date of T2DM incidence was defined as the date of health checkup for those who were ascertained using the health checkup results, and the date of their self-report for those who were ascertained using self-report. Participants were censored when they retired (usual age of retirement is 60 years), except for those who agreed to provide their health information to the researchers after retirement or at death. Participants who provided their home address were periodically (approximately biennially) inquired for their health status and disease histories. Death was ascertained through the employer during and also after employment. In addition, the next of kin was asked to inform us if the participants died in our periodical inquiry. The study protocol was approved by the Ethics Review Committee of Nagoya University School of Medicine, Nagoya, Japan (2007-0504).

### Ascertainment of incident T2DM

T2DM was ascertained using a combination of self-administered questionnaire surveys every 2 years on the medical histories of a subset of illnesses, such as T2DM, and yearly mandated health examinations. Incident T2DM was defined as the following criteria: (1) FBG ≥126 mg/dL (7.0 mmol/L) or glycated hemoglobin ≥6.5% (48 mmol/mol)^10^; (2) use of antidiabetic drugs; or (3) self-reported diabetes. Participants who disclosed a history of T2DM were asked for the full contact information of their attending physicians, and the doctors’ records of their patients’ medical histories were verified.

### NRS measurement

The presence of NRS was evaluated using the following single question item: “Do you feel refreshed after a night’s sleep?” Participants who gave a no response were regarded as having NRS.

### Assessment of baseline variables

Smoking status was divided into three categories: current, former, and never. Alcohol consumption was determined by multiplying the number of times per week they drank alcohol and the amount consumed on each occasion. The results were then converted into grams of ethanol per day. Regular exercise was defined as ≥60 min per day for >12 days per month. Shift work was defined as a schedule that involved rotating shifts, working at night, or doing both. Self-administered questionnaire items were used to assess insomnia symptoms and perceived stress. Difficulty in initiating sleep was assessed by “I find it difficult to fall asleep” and difficulty in maintaining sleep was assessed by “I wake up several times during the night”. Degrees of perceived stress were assessed in four categories and used in the analysis: very much, much, ordinary, and little. Sleep duration was divided into three groups: short sleep (<6 hours), normal sleep (6 to <8 hours), and long sleep (≥8 hours). Family history of diabetes was defined as at least one family member (parents, siblings, or children) having diabetes. BMI was computed using measured weight and height as weight (kg) divided by height squared (m^2^). Venous blood samples were drawn after participants fasted for 8 hours or more. Serum glucose levels were enzymatically determined using the hexokinase method. Follow-up questionnaire surveys were conducted at 6 and 11 years to update lifestyles and medical histories. Height and weight obtained at 6- and 11-year health check-up were used to update the analysis.

### Statistical analysis

Chi-squared test was used to test differences in the proportions of the categorical variables. Continuous variables were summarized as means and standard deviation (SD) and were analyzed using T-test. Kaplan-Meier analysis with log-rank test was applied to investigate the association between NRS and diabetes incidence. Cox proportional hazards models estimated HRs and 95% CIs of incident diabetes in relation to NRS in the following two models. Model 1 was adjusted for age, FBG, BMI, smoking status, alcohol consumption, physical activity, family history of diabetes, and perceived stress. FBG was adjusted as it might confound the NRS-T2DM association (eFigure 1). Model 2 further controlled for all the variables in the model 1 plus sleep-related confounders, including shift work, sleep duration, difficulty in initiating sleep, and difficulty in maintaining sleep.

We included age in the models because the prevalence of NRS may change with age, while the risk of T2DM increases with ageing.^11^ Also, we anticipated that BMI would be an important confounding factor. Furthermore, we performed stratified analyses using model 2 variables by age (<50 years old or ≥50 years old), baseline values of BMI (<25 kg/m^2^ or ≥25 kg/m^2^), shift work (yes/no), and sleep duration (short sleep, normal sleep, or long sleep). Multiplicative interactions of NRS with age category, BMI category, shift work, and sleep duration categories were examined in a model that included the same covariates as model 2.

As a supplementary analysis, NRS and covariates were updated at 6 years’ and 11 years’ follow-up as the time-dependent variables. Variables were updated to newer values, and those missing values were imputed using the last-observation carried forward method.^12^ For the updated NRS analyses, the time-dependent Cox proportion hazard model estimated the HRs and 95% CIs of incident diabetes after adjusting for all time-dependent covariates in model 3. We also did stratified analyses in model 3 using time-dependent variables by age (<50 years old or ≥50 years old). In that age-stratified analysis, for participants who became 50 years or older at the time of updating covariates, their subsequent risk was examined in the older age stratum.

All the statistical analyses were performed by IBM SPSS Statistics 28.0 software (IBM, Armonk, NY, USA). A *P*-value of less than 0.05 was considered statistically significant.

## RESULTS

NRS was complained by 32.0% of the 3,665 participants in our analysis. Mean age of the participants was 47.9 (SD, 7.0) years. Participants with NRS were younger (mean age: 47.0 years) and more likely to be current smokers (38.2%) (Table 1). The degree of physical activity and difficulty in maintaining sleep was lower, but shift work, perceived stress, and short sleep were all higher in NRS participants.

**Table 1.** Demographic and lifestyle characteristics of study participants at baseline by nonrestorative sleep, Aichi, 2002

	RS	NRS	*P* value^a^
*N* (%)	2,491 (68.0)	1,174 (32.0)	
Age, years	48.4 (7.1)	47.0 (6.8)	<0.001
Fasting blood glucose, mg/dL	93.7 (9.5)	93.1 (9.2)	0.053
Body mass index, kg/m^2^	23.3 (2.6)	23.1 (2.6)	0.054
Smoking status, %			<0.001
Current	32.4	38.2	
Former	29.8	24.7	
Never	37.7	37.1	
Alcohol consumption, g/day	20.8 (26.4)	20.2 (28.2)	0.543
Physical activity, %			0.003
Regular exercise	17.2	13.4	
Family history of diabetes, %			0.440
Yes	13.3	14.2	
Perceived stress, %			<0.001
Very much	6.3	15.2	
Much	34.8	49.7	
Ordinary	52.0	32.4	
Little	6.9	2.7	
Shift work, %			0.026
Yes	12.3	15.0	
Sleep duration, %			<0.001
<6 hours	9.6	15.5	
6 to <8 hours	77.8	76.0	
≥8 hours	12.6	8.5	
Difficulty in initiating sleep, %	12.2	13.3	0.355
Difficulty in maintaining sleep, %	22.1	15.9	<0.001

NRS, nonrestorative sleep; RS, restorative sleep.

Continuous variables and categorical variables are reported as mean (standard deviation) and percentage, respectively.

^a^*P* values were obtained using *t*-test and chi-squared test for continuous variables and categorical variables, respectively.

During a median follow-up time of 14.6 years, 421 participants developed T2DM. The crude incidence rate (per 1,000 person-years) of T2DM was 11.2 for participants with NRS, which was higher than that for those without NRS (9.3, log-rank *P* = 0.065) (Table 2). Following adjustment for model 1 covariates, participants with NRS had 33% higher hazard risk for T2DM (HR 1.33; 95% CI, 1.10–1.64). Further adjustment for shift work, sleep duration, difficulty in initiating sleep, and difficulty in maintaining sleep did not significantly change the association (HR 1.36; 95% CI, 1.10–1.67).

**Table 2.** Hazard ratios of type 2 diabetes by baseline nonrestorative sleep, Aichi, 2002–2019

	RS	NRS
*n* of cases/*N*	267/2,491	154/1,174
Crude incidence rate^a^	9.3	11.2
Model 1^b^ HR (95% CI)	1 (ref)	1.33 (1.10–1.64)
Model 2^c^ HR (95% CI)	1 (ref)	1.36 (1.10–1.67)

CI, confidence interval; HR, hazard ratio; NRS, nonrestorative sleep; RS, restorative sleep.

^a^Crude incidence rate per 1,000 person-years.

^b^Model 1 was adjusted for age, fasting blood glucose, body mass index, smoking status, alcohol consumption, physical activity, family history of diabetes, and perceived stress.

^c^Model 2 was further adjusted for shift work, sleep duration, difficulty in initiating sleep and difficulty in maintaining sleep.

The significant positive associations between NRS and T2DM were found in younger (aged under 50 years) participants (HR 1.82; 95% CI, 1.36–2.43) but not in older (50 years or older) participants (HR 1.04; 95% CI, 0.76–1.41) (interaction *P* = 0.025) (Table 3). The presence of overweight, shift work, or sleep duration did not seem to modify the association of NRS with T2DM incidence. Namely, HRs were 1.57 (95% CI, 1.10–2.22) among the overweight and 1.26 (95% CI, 0.96–1.64) among the normal weight individuals (interaction *P* = 0.30). HRs were 1.44 (95% CI, 0.88–2.36) for participant with shift work and 1.35 (95% CI, 1.07–1.71) for those without shift work (interaction *P* = 0.99). HRs were 1.31 (95% CI, 0.73–2.36) for participants with short sleep and 1.37 (95% CI, 1.07–1.75) for those with normal sleep and 1.20 (95% CI, 0.66–2.19) for those with long sleep (interaction *P* = 0.75).

**Table 3.** Incidence rates and hazard ratios of type 2 diabetes according to nonrestorative sleep stratified by age, body mass index, shift work, and sleep duration at baseline, Aichi, 2002–2019

		*n* of cases/*N*	Crude incidence rate^a^	HR (95% CI) Model 2^b^
**Age**				
<50 years old	RS	122/1,288	7.0	1 (ref)
	NRS	91/687	10.1	1.82 (1.36–2.43)
≥50 years old	RS	145/1,203	12.6	1 (ref)
	NRS	63/487	13.1	1.04 (0.76–1.41)
**Body mass index**				
<25 kg/m^2^	RS	166/1,914	7.4	1 (ref)
	NRS	94/916	8.6	1.26 (0.96–1.64)
≥25 kg/m^2^	RS	101/577	15.9	1 (ref)
	NRS	60/258	21.4	1.57 (1.10–2.22)
**Shift work**				
No	RS	224/2,184	8.7	1 (ref)
	NRS	123/998	10.3	1.35 (1.07–1.71)
Yes	RS	43/307	12.8	1 (ref)
	NRS	31/176	16.2	1.44 (0.88–2.36)
**Sleep duration**				
<6 hours	RS	26/238	9.2	1 (ref)
	NRS	28/182	13.0	1.31 (0.73–2.36)
6≤ hours <8	RS	197/1,938	8.7	1 (ref)
	NRS	107/892	10.2	1.37 (1.07–1.75)
≥8 hours	RS	44/315	13.5	1 (ref)
	NRS	19/100	17.7	1.20 (0.66–2.19)

CI, confidence interval; HR, hazard ratio; NRS, nonrestorative sleep; ref, reference; RS, restorative sleep.

^a^Crude incidence rates are expressed as per 1,000 person-years.

^b^Model 2 adjusted for age (if appropriate), fasting blood glucose, body mass index (if appropriate), smoking status, alcohol consumption, physical activity, family history of diabetes, perceived stress, shift work (if appropriate), sleep duration (if appropriate), difficulty in initiating sleep and difficulty in maintaining sleep.

The significant association was not found in time-dependent analyses between updated NRS and T2DM (HR 1.14; 95% CI, 0.92–1.40) (eTable 1). However, in the younger age group, NRS still increased the risk of T2DM (HR 1.77; 95% CI, 1.22–2.57) (eTable 2).

## DISCUSSION

In this cohort of middle-aged Japanese men, the prevalence of NRS was 32.0%. In the previous studies, the prevalence of NRS ranged widely from 2.4% to 42.1% in different countries and using different methods. Previous studies that used a yes-no question to assess the presence of NRS reported the prevalence to be 19.2% to 31.0% in men and 26.3% to 42.1% in women.^6^^,^^13^^,^^14^ Other studies that used frequency-based scale indicated the prevalence to be 6.6% to 28.4% in the United States,^15^^–^^17^ 2.4% to 16.1% in some European countries,^18^ and 4.7% in South Korea.^19^ The prevalence of NRS seemingly varies amongst countries, due possibly to different lifestyles when NRS was evaluated via questionnaires. Additionally, because there are several ways to assess NRS, its frequency varied depending on the evaluation methods.^20^

In the current study, we discovered that, after adjusting for a variety of potential confounding factors, such as age, perceived stress, smoking, and baseline FBG levels, NRS was positively linked with the incidence of T2DM in middle-aged Japanese men. The present finding is consistent with earlier longitudinal research of total 2,291 middle-aged adults conducted in Hong Kong.^8^ We expanded the discovery to a Japanese middle-aged male sample with a longer follow-up period and adjusting for more covariates, including lifestyle variables and perceived stress, which also reportedly increase the risk of T2DM development,^2^^,^^21^ and found that the relationships held true across several subgroups in stratified analyses. Our study’s findings regarding the positive relationship between NRS and the risk of T2DM are also consistent with a prior cross-sectional study conducted in Japan. Namely, NRS was significantly associated with the presence of diabetes (OR 1.159; 95% CI, 1.022–1.314) after adjusting for age, gender, BMI, systolic blood pressure, smoking status, and use of antihypertensive drugs.^11^ Another cohort study conducted in Japan reported that NRS was also linked to an increased risk of diabetes,^9^ consistent with the present study. The present study was able to consider other factors, such as insomnia symptoms and shift work, which could also be related to T2DM incidence.^22^^,^^23^

Although the present finding was observed independent of sleep duration, NRS may indicate the presence of insufficient sleep. Accordingly, we suggest a few potential mechanisms by which NRS might result in T2DM. First, previous studies have revealed an increase in sympathetic nervous system activity after sleep restriction or fragmentation.^24^^–^^26^ Insulin secretion is inhibited by elevated sympathetic nervous system activity, which also develops insulin resistance.^27^ Another possible mechanism between sleep problem and the risk of T2DM may be inflammation. Higher levels of circulating inflammatory markers have been linked to insomnia.^28^ It has been reported that low-grade inflammation is connected to insulin resistance.^29^ Third, sleep deprivation has been linked to alterations in serum cortisol levels and diurnal variations. Many studies have found that cortisol levels elevated in the afternoon and evening, and the magnitude of these increases may be related to insulin resistance.^24^^,^^30^ Moreover, the lack of sleep causes a satiety hormone (leptin) to decrease, an appetite-stimulating hormone (ghrelin) to increase, resulting in increase in hunger and food intake,^31^ which may eventually be related to an increased risk of T2DM.

In the present study, the associations were similar in men with or without overweight (BMI <25 kg/m^2^) or shift work or sleep duration, further indicating independent associations of NRS with T2DM. However, the association did not exist in those aged 50 years or older. In our additional time-dependent analyses, a significant association was not found between updated NRS and T2DM in the whole sample. In contrast, the association between NRS and T2DM was robust in the younger age group even in the updated NRS analysis. The exact reason for not finding the association among the entire study participants is not known; however, since updated analyses would mainly examine a short-term effect of an exposure obtained in the nearest examinations, possible adverse effect of NRS could be captured more efficiently in the analysis using baseline NRS. Also, NRS was more frequently reported by younger participants in the present study, consistent with a previous report.^11^ Participants who previously had NRS might not have reported it later even their T2DM risk became higher due to aging, which might have distorted the association. Further studies could target more on age-related changes of NRS and T2DM risk and their association.

Our study has some limitations. First, the definition of NRS in the present study relies only on a single self-reported item. Although the Nonrestorative Sleep Scale has been proven to be a valid and reliable assessment,^32^ it is not available in Japanese. Hence, our findings might merely indicate a connection between sleep problems and T2DM. Nonetheless, as the primary symptom of unrefreshing sleep was included in the NRS assessment in the present study, the finding indicates a link between NRS and T2DM, especially in younger individuals. Future research should confirm the present finding using standardized, reliable, and valid NRS measurements. Second, only male participants were included in the present study. Men and women, especially those with occupations, may have different sleep-related problems. Further study should include both men and women to confirm our results. Third, we excluded prevalent diabetes at baseline based on FBG and self-reported medication use but not on HbA1c, leaving a possibility that diabetes cases that could have potentially been identified by HbA1c might have existed in the analytic sample.^33^ It is unknown whether the present findings were influenced by this limitation, and further studies are necessary for confirmation. Finally, the study subjects were civil servants working in a single prefecture in Japan. Although they are less heterogeneous than the general Japanese population, which is advantageous in assessing the association, they may have better socioeconomic conditions and might be more health-conscious than the ordinary Japanese, and the finding may only apply to similar population.

### Conclusion

The present study suggested that NRS increased the risk for developing T2DM in middle-aged Japanese male workers independent of a number of lifestyle factors, perceived stress, and sleep problems, as well as the baseline BMI and FBG. The associations were similar in men with or without baseline overweight, shift work, and sleep duration. However, the association in older men aged 50 year or older was not evident. Future study about NRS in Japan should consider applying a reliable and valid Japanese version of Nonrestorative Sleep Scale. Furthermore, it should be examined if the association would be observed in different populations, including women or non-working individuals.

## SUPPLEMENTARY MATERIAL

eTable 1. Hazard ratios of type 2 diabetes according to updated nonrestorative sleep, Aichi, 2002–2019

eTable 2. Incidence rates and hazard ratios of type 2 diabetes according to updated nonrestorative sleep stratified by age, Aichi, 2002–2019

eFigure 1. Directed Acyclic Graph (DAG) for Nonrestorative Sleep and Type 2 Diabetes
