# Supplementary material for: Nonrestorative Sleep and Type 2 Diabetes Incidence: The Aichi Workers’ Cohort Study
Source: J Epidemiol. 2024 Sep 5;34(9):428–33. doi: 10.2188/jea.JE20230184 (PMC11330709; doi:10.2188/jea.JE20230184)
Supplement: Supplementary file 1 [file je-34-428-s001.pdf]

eTable 1. Hazard ratios of type 2 diabetes according to updated nonrestorative sleep, Aichi, 2002–2019

	RS	NRS
Crude incidence rate[a]	9.7	10.2
Model 3[b] HR (95% CI)	1 (ref)	1.14 (0.92–1.40)

CI, confidence interval; HR, hazard ratio; NRS, nonrestorative sleep; RS, restorative sleep.

[a]Time-dependent crude incidence rate per 1000 person-years within follow-up intervals.

[b]Model 3 adjusted for baseline age, updated fasting blood glucose, body mass index, smoking status, alcohol consumption, physical activity, family history of diabetes, perceived stress, shift work, sleep duration, difficulty in initiating sleep and difficulty in maintaining sleep.

eTable 2. Incidence rates and hazard ratios of type 2 diabetes according to updated nonrestorative sleep stratified by age, Aichi, 2002–2019

	RS	NRS	RS	NRS
**Age**	<50 years old		≥50 years old	
Crude incidence rate[a]	6.6	8.9	11.8	11.2
Model 3[b] HR (95% CI)	1 (ref)	1.77 (1.22–2.57)	1 (ref)	0.94 (0.72–1.21)

CI, confidence interval; HR, hazard ratio; NRS, nonrestorative sleep; ref, reference RS, restorative sleep.

[a]Time-dependent crude incidence rate per 1000 person-years within follow-up intervals.

[b]Model 3 adjusted for updated fasting blood glucose, body mass index, smoking status, alcohol consumption, physical activity, family history of diabetes, perceived stress, shift work, sleep duration, difficulty in initiating sleep and difficulty in maintaining sleep.

eFigure 1

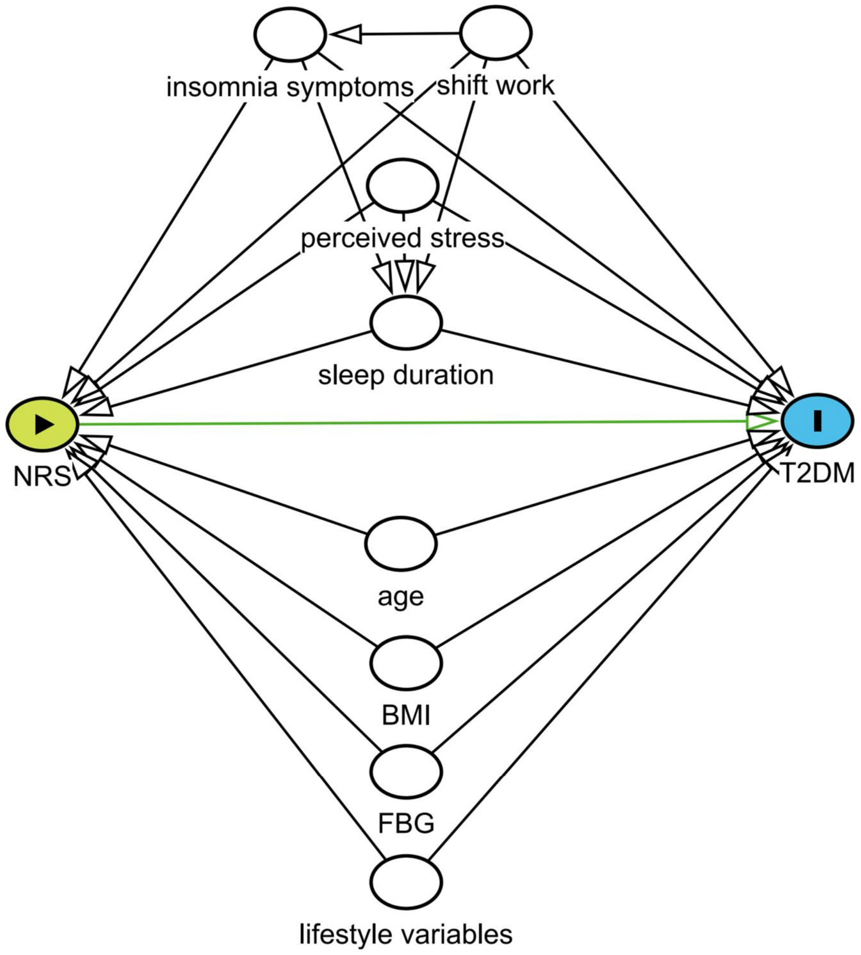

Directed Acyclic Graph (DAG) for Nonrestorative Sleep and Type 2 Diabetes

Lifestyle variables include smoking status, alcohol consumption, and physical activity; insomnia symptoms include difficulty in initiating sleep and difficulty in maintaining sleep. BMI, body mass index; FBG, fasting blood glucose; NRS, nonrestorative sleep; T2DM, type 2 diabetes.
